# The Washout Resistance of Bioactive Root-End Filling Materials—A Systematic Review

**DOI:** 10.3390/jcm14072446

**Published:** 2025-04-03

**Authors:** Joanna Falkowska-Ostrowska, Włodzimierz Dura

**Affiliations:** Department of Preclinical Conservative Dentistry and Preclinical Endodontics, Pomeranian Medical University in Szczecin, Al. Powstancow Wlkp. 72, 70-111 Szczecin, Poland; wlodzimierz.dura@pum.edu.pl

**Keywords:** washout resistance, dental materials, root-end filling materials, bioceramics

## Abstract

When performing an apicoectomy avoiding the microleakage is desired. That is why materials used for this procedure should be resistant to washout. Washout refers to the tendency of freshly prepared materials to disintegrate upon contact with fluids. **Background**/**Objectives**: The aim of this paper is to provide a literature review on the washout resistance of bioactive root-end filling materials. **Methods**: This systematic review was conducted following the PRISMA 2020 guidelines. International databases (PubMed, Google Scholar, ScienceDirect, and Wiley Online Library) were searched, and articles published in the last 20 years were selected for analysis. The following keywords were used “antiwashout”, “washout resistance”, “washout in dentistry”, “root-end filling materials”, “calcium silicate-based cements”, “bioceramic materials”. A total of 6 in vitro studies that met all the inclusion criteria were included in the analysis. The overall risk of bias was low in all six studies. **Results**: Most tested bioceramic materials are Endocem, Capasio, and Ceramicrete-D. Generex-A, MTA-Plus, MTA-AW, Bioaggregate, and MTA HP usually demonstrate very good washout resistance. ProRoot and MTA Angelus performed differently depending on the test; however, generally they showed good washout resistance. The Biodentine material showed significant washout, and requires further research. There is still a lack of a unified method for washout evaluation in dentistry, which makes it difficult to compare different studies. **Conclusions**: The study the confirmed excellent washout resistance of EndoCem, Capasio, Ceramicrete-D, Generex-A, Bioaggregate, MTA-Plus, and MTA HP. ProRoot, MTA-Angelus, and MTA Angelus White exhibited lower washout resistance. Biodentine shows poor washout resistance, and requires further research. A unified method for assessing washout would be beneficial for comparing different studies.

## 1. Introduction

In recent years, contemporary endodontics has witnessed unprecedented advances in technology and materials. However, endodontic treatment still presents significant challenges in cases where conventional root canal treatment does not yield the desired results or when reinfection occurs. Apicoectomy (root apex resection) may be recommended. This surgical procedure involves a soft tissue incision, followed by removal of localized diseased tissue, resection of the root apex, root-end preparation, and retrograde filling [1]. For many years, apicoectomy has been performed only on single-rooted teeth. However, owing to the progress in apical surgery involving the introduction of Cone Beam Computed Tomography (CBCT), dental operating microscopes, and ultrasonic techniques, it has become possible to perform this procedure on premolars and molars [2]. These devices increased the precision of the procedure, significantly reduced its invasiveness, and had a positive impact on the effectiveness of resection. Studies have shown that the use of a microsurgical approach provides better clinical results [3,4]. Compared with the traditional approach, the microsurgical approach results in better healing of soft tissue and fewer postoperative complications [1]. A very important step in performing root apex resection is retrograde filling of the root canal. Currently, retrograde root canal filling is routinely performed during apical surgery [2,5]. In the past, retrograde filling was not performed; if the canal was correctly filled, it was assumed to be an expensive, time-consuming, and difficult procedure [6]. While there is consensus regarding routine root-end filling after root apex resection, the choice of material, which would ensure a tight apical seal from the periapical tissues, is still being discussed. A long time ago, a commonly used material was an amalgam that was used for the first time for this purpose towards the end of the 19th century [7]. The use of this material is currently questioned due to its poor sealing properties and lack of biocompatibility [8]. Previously used glass ionomer cements, resin-modified glass ionomer cements, and cermet ionomer cements are no longer used. While materials based on the zinc oxide-eugenol cement Intermediate Restorative Material (IRM, Dentsply Sirona, York, PA, USA) or Superethoxy Benzoic Acid (Super EBA, Harry J. Bosworth Co., Skokie, IL, USA) are still used by some dentists [9]. For over a quarter of a century, Mineral Trioxide Aggregate (MTA) has been commonly used and is currently the material of choice. MTA is a so-called hydraulic calcium silicate cement [10]. Original material ProRoot MTA (Dentysply Maillefer, Tulsa, OK, USA) sets for a relatively long time (up to 4 h), while newer materials set faster, usually 12–15 min, depending on the manufacturer (e.g., MTA Angelus, Angelus, Brazil). MTA is difficult to apply, but its contact with fluids, including blood, does not interfere with its setting. It is recommended as a root-end filling material due to its biocompatibility and bioactivity [11]. Freshly mixed MTA cement may be washed out as a result of contact with fluids, e.g., physiological saline solution used for rinsing the resection crypt to remove any residues of material or blood flowing into a niche after the procedure [8,12]. New materials have recently been introduced to the market, bioceramics, which unlike MTAs, do not cause discoloration of the tooth tissue (instead of bismuth oxide, they contain tantalum or zirconium oxide), are biocompatible, and according to the manufacturer’s information, are characterized by washout resistance [13].

Washout is the tendency of freshly prepared cement paste to ‘disintegrate upon early contact with blood or other fluids’ [14]. Grech et al. [15] created a mathematical formula that describes this phenomenon, in which ‘washout (or loss of mass of the sample) was expressed as a percentage of the initial mass of the sample and calculated using Equation’. The term washout comes from engineering science and is a term used to describe the washing out of material from freshly mixed cement by fluids. It is usually used to measure the washout resistance of underwater concrete [16]. The difference between washout and solubility is that in the process of analyzing solubility, the material can be tested after it sets, and the time of immersion of the sample is longer [17].

Washout may have an impact on the result of the treatment, since the loss of the filling material may cause microleakage. This is one of the reasons why this property has been studied by researchers over the years. However, to the best of the authors’ knowledge, there has been no effort to sort and summarize studies analyzing the washout resistance of bioactive root-end filling materials. Therefore, the aim of this study was to analyze the currently available literature describing the washout resistance of bioactive root-end filling materials and provide some useful comparisons of materials that may be helpful in clinical procedures. The authors also aim to present the different research methodologies used in various studies and to demonstrate the need for further research.

## 2. Materials and Methods

### 2.1. Methodology

#### Study Design

The protocol of this study was based on the Preferred Reporting Items for Systematic Reviews and Meta-analyses (PRISMA) 2020 checklist (Page et al., 2021) [9,18] and the population, intervention, comparison, outcome (PICO) strategy (Table 1) [19]. Although the PRISMA protocol was used, the review was not registered in any database, as most of the work was performed manually.

The following research question was developed:

Are bioactive root-end-filing materials resistant to washout based on research that used in vitro tests?

The following selection criteria were established:

Inclusion criteria

1. Studies that were testing bioactive root-end filling materials
2. Studies that were testing washout resistance in dentistry
3. Studies that were in English
4. In-vitro studies

Exclusion criteria

1. Studies that tested only solubility, not washout
2. Nonhuman studies
3. Reviews and book chapters

### 2.2. Search Strategy in the Databases

International databases (PubMed, Google Scholar, ScienceDirect, and Wiley Online Library) were searched by two independent reviewers, and articles published in the last 20 years were selected for analysis. The following keywords were entered: “antiwashout” OR “washout resistance” OR “washout in dentistry” AND “root-end filling materials” OR “calcium silicate-based cements” OR “bioceramic materials” OR “bioceramic” OR “bioceramics” OR “bioactivity” OR “bioactive.” A manual search of dentistry journals in the journal section of Pomeranian Medical University in Szczecin was also conducted. The research was carried out until December 2024.

### 2.3. Study Selection

Initially, all duplicate studies were manually removed and considered only once. Further selection of studies was based on the analysis of titles and abstracts. Next, the full texts of the selected studies were read, and studies that met the inclusion criteria were included in the review.

### 2.4. Data Extraction from the Eligible Studies

After selecting six eligible articles, two independent reviewers extracted the following data: (a) author and year of publication, (b) tested materials, (c) testing methods, (d) test duration, (e) used solution, (f) method of evaluation, (g) test results, and (h) conclusions.

### 2.5. Risk of Bias Assessment

The risk of bias was assessed using the Joanna Briggs Institute (JBI) tool [20].

As recommended by PRISMA [19], two examiners assessed each study based on the criteria used to rank the risk of bias. A low risk of bias was detected when positive answers were >70%. All eligible studies had a low risk of bias, showing a high percentage of positive answers to the questions of the JBI tool (Table 2).

## 3. Results

### 3.1. Search Details

**Figure 1:**
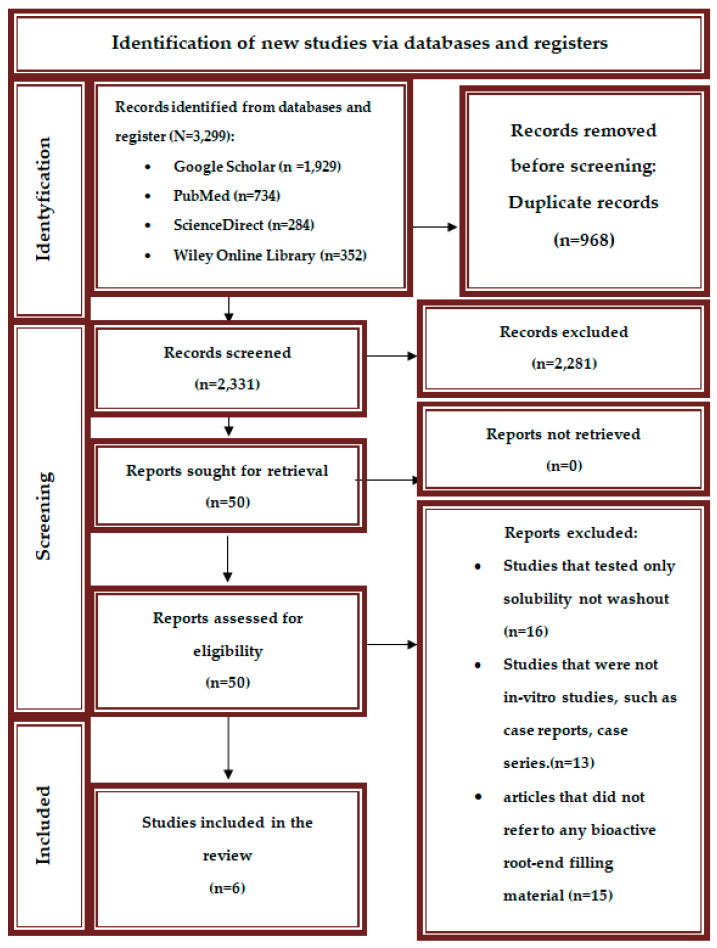
Preferred reporting items for systematic reviews and meta-analyses (PRISMA) flow diagram of the study screening procedure and selection.

Online databases (PubMed, Google Scholar, ScienceDirect, and Wiley Online Library) and the library of the Pomeranian Medical University in Szczecin were searched, and a total of 3299 papers were found. After removing duplicate studies, 2331 articles remained. Next, the titles and abstracts of the articles were screened. Fifty studies were selected for full-text evaluations. Researchers excluded 16 studies that tested only solubility, not washout, 13 studies that were not in vitro studies, and 15 articles that did not refer to any bioactive root-end filing material. Using the exclusion and inclusion criteria, six studies were eligible for this analysis. The details are shown in the flow diagram of the study screening procedure and selection in Figure 1.

### 3.2. Washout in Dentistry

The success of endodontic treatment is based on the eradication of infection from the root canals and a tight seal. In some cases, despite our efforts, we may still observe some apical changes or other complications that will create a need for endodontic surgery. When performing root-end surgery, it is important to choose the appropriate sealing material. Reparative materials for apicoectomy should have all or most of the following properties: adhere to and seal the root, be dimensionally stable, radiopaque, induce healing of periradicular tissue, be biocompatible, and be resistant to washout. Washout, expressed as the loss of mass of the sample, can be caused by washing the cavity after the procedure or by other fluids that come into contact with the material. This leads us to the conclusion that washout may have a considerable impact on the result of the procedure, as the loss of the filling material can cause microleakage. This literature review reveals the need to create a standardized method for measuring the washout resistance of dental materials, which can provide results that will be reflected in the clinic. There is a wide range of materials available for root-end surgery. We have materials that are well known for years now, like Biodentine (Septodont, St Maur-des-Fosses, France), ProRoot MTA (Maillefer Dentysply, Tulsa, OK, USA), and MTA-based cements, as well as some newly developed materials that are mostly based on bioceramics, such as Well Root (Vericom, Chuncheon, WR, Republic of Korea), TotalFill BC (FKG, La Chaux-des-Fondsm, Switzerland), iRoot (Innovative Bioceramix, Vancouver, BC, Canada), NeoPutty (Avalon Biomed, Bradenton, FL, USA), BIO-C Repair (BCR, Angelus, Londrina, PR, Brazil) and One-Fil (Mediclus, Cheongju, OF, Republic of Korea) [26]. Washout impacts not only endodontics but also surgery, as the washout of bone substitutes is an undesirable occurrence [27].

### 3.3. Standardized Washout Test in Engineering

For underwater concrete testing, CRD C61 and MC-1 tests were used. The CRD C61 (the plunge test) probably originated in Belgium, then it was adopted by the US Corps of Engineers into its current form, and the MC-1 (the new spray test) was developed at the University of Paisley. CRD C61 test uses a basket with holes of 20 mm in diameter, which is filled with concrete and then immersed five times in water. The MC-1 test produces quantitative results. First, the mass of a sample of concrete is determined, and then it is placed into the test apparatus, where it is washed out with water. To spray test 1 kg of fresh concrete is used, then it is put in the mold and placed on a baseplate. Next, the mold is removed, and the sample on the plate is placed on a frame suspended from an electronic balance, so the test is controlled and recorded by the computer. The sample is washed for 4 min. The result is calculated based on the percentage of mass lost compared to the original mass [28]. These tests require considerable amounts of tested materials; therefore, these methods cannot be used to test dental materials without modifications.

### 3.4. Tested Materials

The rapid development of dental materials has generated a wide variety of products on the market. Companies are trying to outdo each other in creating new materials that meet the increasingly demanding requirements of dentists. Biocompatibility has become a desired property, and a number of products called bioceramics have emerged recently, providing us with many new opportunities to treat our patients even better. An increasing number of manufacturers have mentioned washout resistance as another advantage of their products. This property is especially important for products used for direct pulp capping, root resorptions, perforations, apexification, and retrograde fillings. The materials used for these purposes and mentioned in the articles used for the review are detailed below. These materials are described in more detail as they have been tested in the literature described. However, it should be noted that many other materials from this group are available on the market today. This encourages further research.

#### 3.4.1. EndoCem MTA (Maruchi, Wonju-si, Republic of Korea)

Endocem is an MTA-type material. It is based on CaO, Al_2_O_3_, SiO_2_, MgO, Fe_2_O_3_, SO_3_, TiO_2_, H_2_O/CO_2_, and bismuth oxide. Setting times are quick, with an initial setting time of 2 min (±30 s) and final setting time of 4 min (±30 s). Due to its high biocompatibility, it is used in apical retrofilling, root perforations, and vital pulp therapies. The material does not cause discoloration of the tooth tissue, is non-miscible with liquid components, and maintains its integrity when in contact with blood. When washout was tested, it showed higher washout resistance than the classical “long-setting” MTA material [29].

#### 3.4.2. ProRoot MTA (Maillefer Dentysply, Tulsa, OK, USA)

This material is based on mineral trioxide aggregate and can be used as a sealer, apical filler, or for closing perforations. It has good antibacterial, osteogenic, and osteoconductive properties. ProRoot’s initial setting pH is 11.6 and final setting pH is 11.7. The initial setting time is 78 min (±5 min) and final setting time is 261 min (±21 min). The material sets in moisture and has good hydrophilic properties [30].

#### 3.4.3. Ceramicrete-D (Tulsa Dental Specialities/Argonne National Laboratory, Argonne, IL, USA)

It is a self-setting phosphate ceramic developed at Argonne National Laboratory. Ceramicrete is biocompatible, radiopaque, and can be used as a root-end filling material. The initial setting time is 6 min and the final setting time is 12 min. This ceramic-based material sets underwater with minimal washout [30].

#### 3.4.4. Capasio (Primus Consulting, Bradenton, FL, USA)

The material composed primarily of bismuth oxide, dental glass, and calcium alumino-silicate with silica and polyvinyl acetate-based gel. It can be used as a root-end filling material and can penetrate the dental tubules [30].

#### 3.4.5. Generex-A (Dentsply Tulsa Dental Specialities, Tulsa, OK, USA)

This is a calcium silicate-based material, similar to MTA, but mixed with specific gels instead of water. Generax A is intended for retrograde filling and perforation repair. Material supports primary osteoblast growth, has compressive strength and radiopacity, and has superior resistance to washout [26].

#### 3.4.6. Biodentine (Septodont, St Maur-des-Fosses, France)

It contains tricalcium silicate, calcium carbonate, zirconium oxide, and calcium chloride and was designed as a “dentine replacement”. This material can be used for the treatment of root perforations, resorptions, apexification, retrograde fillings, pulp, capping procedures, or as a dentine replacement under composite restoration. Its pH is 12.5, working time is up to 6 min, and the final setting time is 10–12 min. Biodentine is also radiopaque [31].

#### 3.4.7. BioAggregate (Innovative Bioceramix, Vancouver, BC, Canada)

This calcium silicate cement has quantities similar to those of MTA cement. It has a good marginal seal, superior adhesion, high strength, excellent sealing, sets in the presence of moisture, and is easy to handle. This bioceramic cement has antibacterial properties, is non-toxic, and is aluminum-free. The main indications include the treatment of root perforations, resorptions, root-end fillings, apexification, and pulp capping. It is claimed to be the most biocompatible material among root-end filling materials [30].

#### 3.4.8. MTA-Angelus (Angelus, Londrina, PR, Brazil)

MTA-Angelus is composed of calcium carbonate, calcium silicate, calcium aluminate, and barium zinc phosphate. This bioceramic reparative cement contains 80% Portland cement and 20% Bi_2_O_3_. It is an endodontic cement that is biocompatible, promotes osteogenesis, and has been widely used for retrofilling in endodontic surgeries, resorption, treatment of root canal perforations, pulp capping, apexification, and apexogenesis. The setting time is 15 min [32].

#### 3.4.9. MTA-Plus (PrevestDenpro, Jammu, India Avalon Biomed Inc., Bradenton, FL, USA)

The powder is mixed with purified water. The powder contains calcium oxide, silicon oxide, and iron oxide. Sodium oxide, aluminum oxide, potassium oxide, bismuth oxide, magnesium oxide, zirconium oxide, and calcium phosphate. The compound is cured in 6 to 10 min. The high content of calcium ions remineralizes the tooth tissue. Tissue regeneration after wall perforation and intracanal resorption is supported by silicon- and calcium compounds. This product is especially recommended for pulp amputation, direct coating of the pulp, root canal wall perforation, and intracanal resorption [33].

#### 3.4.10. MTA HP (Angelus, Londrina, PR, Brazil)

MTA HP is a bioceramic repair cement with the addition of an organic plasticizer in the formula, aiming to provide a putty-like consistency at the end of manipulation. It has the same indications as MTA Angelus. The final setting time is 85 min [34].

### 3.5. Washout Tests

Despite the fact that a number of researchers have described different methods for evaluation of washout resistance of dental materials, there is still no standardized method in the literature. There are a couple of known and standardized tests that can be used for the assessment of the washout resistance of concrete, such as the CRD C61 and MC-1 tests [17]. However, these tests are created for larger volumes of materials than those used in dentistry, and the specifications of endodontic treatment are different; therefore, we cannot use one of these tests to measure the resistance to washout of dental materials without adjustments. A number of researchers have evaluated different methods for measuring the washout resistance of dental materials. Sometimes, adapting methods used for evaluating the resistance of freshly mixed concrete to washout in water, such as CRD-C 661-06, for assessment the washout resistance of root-end filling materials. To adapt this test to smaller quantities of material, Formosa et al. (2013) [24] used a test tube with an internal diameter of 14.5 mm. The container was then filled to a height of 120 mm with distilled water or HBSS at room temperature (23 °C). The next step was the construction of a cylindrical container with a 9.0 mm diameter and a height of 17 mm from two pieces of woven brass mesh. Before starting the examination, the empty container was weighed. A quantity of material was placed in the cylinder, and the mass of the cement in the cylinder was calculated. Then, researchers performed three drop cycles for each specimen. The results were calculated using the equation that describes the washout as the percentage loss of the initial mass of the sample compared to the mass of the sample before the initial drop [24].

Grech et al. (2013) [15] likewise used the basket drop method. To perform this test, a test tube with an internal diameter of 14.5 mm was required, and the tube was filled with distilled water to a height of 120 mm. Another part of the test setup was a woven brass mesh cylinder with a diameter of 9.0 mm and a height of 17 mm. The first step was to weigh the empty mesh cylinder, which was then filled with 1 g of the test material and reweighed. The cylinder was released just above the surface of the water in the tube and allowed to sink unhindered. Subsequently, it was left at the bottom for 15 s, then removed from the fluid in about 5 s and allowed to drip for 2 min. The cylinder was then patted dry with absorbent paper and weighed again. For each material, the entire procedure was repeated three times [15].

Other investigators have created unique tests for specific research. Porter et al. (2010) [23] created a method in which artificial root-ends were created in plastic blocks, and water was sprayed perpendicular to the cavosurface; in this study, the samples were photographed before and after washout testing and evaluated by two blinded evaluators.

In another study [35], a Teflon mold filled with material was placed into FBS, and SEM was performed. It is worth mentioning that some more sophisticated methods used to assess the solubility could be used to assess washout; the only problem is the time after mixing the cement, which is needed for scanning and other steps, since washout tests are performed on freshly mixed material, while solubility tests can take much more time because these tests are evaluating fully cured materials.

In the last study that was analyzed [25], the authors created their own washout test. Washout resistance was assessed using artificial root-end preparations 1.2 mm × 3 mm in plastic blocks. In total, 150 samples (30 for each material) of freshly prepared material were placed in artificial root-ends prepared in plastic blocks. Then, two experiments were conducted. In the first experiment, 75 samples (fifteen samples of each material) were photographed under a microscope immediately after the material application was completed and rinsed with 5 mL of saline for 15 s using a syringe and a needle with a diameter of 0.8 mm and a length of 12 mm. After completing the simulation of rinsing the bony crypt, the models were immersed in saline at 34 °C for 15 min (reflecting the blood flooding the crypt). In the next step, the models were removed and carefully dried to avoid damaging the fillings, and then photographed again using a microscope. In the second experiment, the same number of samples was used as in the first experiment, with the difference that in addition to photographing the samples using a microscope, the surface of the fillings was scanned using a 3D dental scanner (KaVo ARCTICA AutoScan; KaVo, Biberach, Germany). Scanning and photography took 3 min. The samples were rinsed as in Experiment 1 and immersed in warm saline for 15 min, after which the models were removed, photographed under a microscope, and scanned again. The obtained scans were then superimposed on each other to obtain a color model in the form of a depth map (Table 3).

### 3.6. Washout of Materials in Analyzed Studies

Jang et al. [21] showed that Endocem exhibit superior washout resistance compared to ProRoot. Nevertheless, Smith et al. [22] indicated that irrigating ProRoot with EDTA or BioPure results in only minor volume reduction of the set MTA. Porter et al. [23] compared White MTA ProRoot with newly developed cements

(Capasio, Ceramicrete-D, and Generex-A). The washout test showed that the new materials were resistant to rinsing and that the WMTA samples had significant washout, losing as much as 1 mm of depth from the artificial root-end preparation. Formosa et al. described the results by calculating the percentage weight loss of the cement. The results showed a mass loss of 2–7% for Portland cement, 0.4–4% for MTA-Plus, 0.9% for MTA-AW, 5–10% for MTA Angelus, and 0% for IRM and amalgam. In a study conducted by Grech et al. [15], it was shown that radiopacified tricalcium silicate, Bioaggregate, and IRM exhibited very low washout, whereas Biodentine showed a very high washout tendency. A concerning washout of Biodentine was also described by Falkowska et al. [25]. The study also showed that rinsing and immersing the samples in a liquid simulating blood immediately after filling the root end did not cause the disintegration of fillings made of IRM, MTA Angelus White, EndoCem MTA Zr, and MTA HP (Table 4).

## 4. Discussion

In recent years, bioceramics have gained significant attention in the field of dentistry. Many reviews have described various issues related to these materials. Küçükkaya wrote a narrative review that focused on the clinical applications of calcium silicate-based materials and described their various properties, including setting time and solubility [36]. Ortega et al. compared bioceramics with traditional biomaterials used as endodontic sealers in terms of physicochemical and biological properties [37]. The effects of bioceramic sealers on dentinal tubule penetration and antimicrobial effectiveness were described in a meta-analysis conducted by Seron et al. [38]. In a systematic review and network meta-analysis by Komora et al. [39], failure rates in vital pulp treatment were compared between traditional MTA and novel calcium silicate-based cements. Despite the frequent discussion on the topic of bioactive materials in literature reviews in recent years, none of the authors have described a review focusing on the washout of bioactive materials used for retrograde root apex filing.

This systematic review aimed to investigate the washout resistance of bioactive materials available on the market to provide helpful guidance in selecting the ideal retrograde obturation material. Nevertheless, the limited number and limitations of the included studies must be considered.

Materials used for retrograde root canal filling should be resistant to washout. Washout of retrograde filing during setting, due to rinsing the resection crypt with saline or contact with body fluids (e.g., blood), may cause microleakage. In such cases, persistent intracanal infection impedes the healing of periodontal tissues after apicoectomy, and after healing, leads to the development of de novo inflammatory changes [1].

Based on the present findings, it was possible to verify that most of the tested materials (EndoCem [21,25], Ceramicrete-D [23], Generex-A [23], Capasio [23], Bioaggregate [15], MTA-Plus [24], MTA HP [25]) showed very good washout resistance.

ProRoot [21,22,23] and MTA Angelus/MTA Angelus white [24,25] performed slightly worse in these studies. The relatively good resistance of MTA Angelus to washout has been confirmed by numerous clinical studies, both prospective and retrospective, in which a relatively high percentage of positive treatment results was recorded, ranging from 84 to 96% [4,40]. ProRoot was the most tested material and was described in three different studies. Jang et al. compared it with EndoCem and showed that ProRoot exhibits significantly lower washout resistance (*p* < 0.05). Smith et al. evaluated the surface of the White ProRoot MTA after the use of ethylenediamine tetra-acetic acid (EDTA) and BioPure MTAD and reported only minor volume reductions of the cement. In another study, Porter et al. stated that WMTA samples were characterized by significant washout of 80–100% of the margin and a depth loss of up to 1 mm. MTA Angelus fared the worst among the materials tested by Formosa et al., with 5–10% mass loss. However, Falkowska et al. showed only slightly greater washout than that of EndoCem and MTA HP. Biodentine was tested in two different studies [15,25], and in both, it showed poor washout resistance, which is concerning and requires further research (Table 5).

A clinical limitation of this systematic review is the use of in vitro studies only. In vitro studies are highly focused, enabling the deduction of mechanisms of action and control of many confounding variables. However, the weakness of this type of study is the uncertainty that the effects observed in vitro would be identical to those observed in the clinical setting. The methodology closest to clinical settings was used by Falkowska et al. [25]. Based on the studies described above, we can suggest some clinical recommendations. During the filling of the prepared root apex, gentle rinsing of the resection cavity is recommended; bleeding during the apex resection procedure is also responsible for the disintegration of the filling [24]. Therefore, cements used for retrograde filling should be resistant to washout. This resistance increases with time, although it should be remembered that it is difficult to guarantee dryness during the procedure for several minutes. Therefore, selecting the appropriate material is crucial.

Researchers have used different solutions in their studies. Jang et al. [21] used physiologic saline, 2.5% sodium hypochlorite (NaOCl), and 2% chlorhexidine (CHX), showed in their study that the washout scores were significantly lower in the NaOCl-treated group compared to the saline- and CHX-treated groups (*p* < 0.05). Smith et al. [22] used 17% EDTA and BioPure MTAD, and they stated that more material was removed by BioPure MTAD, although these minor depth changes are unlikely to cause clinical concern. Water was used by Porter et al. [23] and Grech et al. [15]. Formosa et al. [24] rinsed the material with distilled water and HBBS and found no significant differences between the use of water and HBSS as washout media for the same material. Falkowska et al. [25] used saline. Currently, there is insufficient data in the literature to clearly assess the effect of the type of substance used for irrigation on washout.

The main limitation of this study was the limited number of studies conducted on the analyzed topic, which was associated with a relatively small research group. Including articles published in other languages may help avoid language bias. Excluding studies not published in English may result in the omission of some data and potentially alter the conclusions of the review. The lack of a standardized washout testing method makes it difficult to compare the results (Table 6). This complicates the comparison of results from different studies and may potentially lead to errors. Therefore, it would be beneficial to conduct an extended review in the future that collects articles without language restrictions.

## 5. Conclusions

According to many studies included in this review, most bioactive root-end filling materials exhibit excellent washout resistance (EndoCem, Capasio, Ceramicrete-D, Generex-A, Bioaggregate, MTA-Plus, and MTA HP). ProRoot, MTA-Angelus, and MTA Angelus White show lower washout resistance. Studies investigating washout resistance of Biodentine have shown that it is prone to washout. Thus, further studies are required to confirm these findings.

Despite the fact that a number of researchers have described different methods for the evaluation of washout resistance of dental materials, there is still no standardized method in the literature. This literature review reveals the need to create a unified method for measuring the washout resistance of dental materials, which can provide results that will be reflected in the clinic.

## 6. Future Directions

Some studies have indicated that Biodentine is susceptible to washout, which is concerning and requires further investigation.

The introduction of a unified methodology for testing washout resistance in dentistry would be of great importance for future research.

## Figures and Tables

**Table 1:** Description of the PICO strategy.

Acronym	Definition	Description	Description of the Components of PICO in This Systematic Review
P	Patient, population, or problem	Can be a patient, a group of patients with a particular condition, or a health problem. What is the nature of the problem?	Washout of bioactive root-end filling materials
I	Intervention	Represents the intervention of interest. What is the primary solution being considered?	Root-end filling materials that are washout resistant
C	Control or comparison	Defined as a standard intervention, the most used intervention, or no intervention	Comparison of different materials
O	Outcome	Expected result	Highlighting materials that are less prone to washout and therefore provide a better seal

**Table 2:** Risk of bias of the included studies, according to the Joanna Briggs Institute (JBI) tool for assessing the risk of bias. Q1. Were the criteria for inclusion in the sample clearly defined? Q2. Were the study subjects and the setting described in detail? Q3. Was the exposure measured in a valid and reliable manner? Q4. Were objective, standard criteria used for the measurement of the condition? Q5. Were confounding factors identified? Q6. Were strategies to deal with confounding factors mentioned? Q7. Were the outcomes measured in a valid and reliable manner? Q8. Was an appropriate statistical analysis used? +—Yes.

Study	Q1	Q2	Q3	Q4	Q5	Q6	Q7	Q8	% Yes	Risk
Jang et al., 2013 [21]	+	+	+	+	+	+	+	+	100	Low
Smith et al., 2007 [22]	+	+	+	+	+	+	+	+	100	Low
Porter et al., 2010 [23]	+	+	+	+	+	+	+	+	100	Low
Grech et al., 2013 [15]	+	+	+	+	+	+	+	+	100	Low
Formosa et al., 2013 [24]	+	+	+	+	+	+	+	+	100	Low
Falkowska et al., 2023 [25]	+	+	+	+	+	+	+	+	100	Low

**Table 3:** Washout Tests.

Author	Test Type	Test Duration	Solution	Tested Material	Evaluation
Jang et al., 2013 [21]	Material was placed into acrylic molds, on saline-moisted oasis, then exposed to solution under gentle shaking	5 min + shaking, 24 h in incubator at 95 ± 5% humidity and 37 °C	Physiologic saline, 2.5% sodium hypochlorite (NaOCl), 2% chlorhexidine (CHX)	EndoCem (Maruchi, Republic of Korea), ProRoot MTA (Tulsa Dental Specialities, USA)	Scoring scanning electron microscope (SEM) images, 3 independent dentists according to the listed criteria
Smith et al., 2007 [22]	MTA condensed into cylindrical wells created in plexiglass platforms	EDTA 5 min, MTAD 1, 3 or 5 min.	17% EDTA, BioPure MTAD,	ProRoot white MTA (Dentsply Tulsa Dental, USA)	3-D profilometry
Porter et al., 2010 [23]	novel test developed for washout resistance	5 mL with flow rate of 0.33 mL/s	Water	White MTA ProRoot (Dentsply Company, USA), Capasio (Primus Consulting, USA), Ceramicrete-D (Tulsa Dental Specialities, USA), Generex-A (Dentsply Tulsa Dental Specialities, USA)	Using photos to determine the percentage of margin remaining by two independent evaluators.
Grech et al., 2013 [15]	novel basket-drop method	The cylinder was left at the bottom for 15 s then brought out of the water in 5 ± 1 s and allowed to drip for 2 min. Three drop cycles per specimen.	Water	Tricalcium silicate cement (Mineral Research Processing, France) replaced with 20% zirconium oxide (ZrO_2_; Sigma–Aldrich, Germany)—TCS-20-Z; BiodentineTM (Septodont, France); BioaggregateT (Verio Dental Co., Ltd., Canada); Intermediate restorative material (Dentsply DeTrey, Germany)—IRM;	Loss of mass of the sample is calculated using Equation.
Formosa et al., 2013 [24]	a quantitative method, modified CRD-C 661-06	The cylinder was left at the bottom for 15 s, then brought out of the water in 5 ± 1 s and allowed to drip for 2 min. Three drop cycles per specimen.	Distilled water, HBBS	MTA-Plus (Avalon Biomed, India), Portland cement (PC; CEM 1, 52.5 N; LaFarge Cement, UK), MTA-Angelus (Angelus, Brazil), IRM (Dentsply, Germany), amalgam (AB Ardent, Sweden).	Loss of mass of the sample is calculated using the Equation.
Falkowska et al., 2023 [25]	novel test developed for washout resistance	The rinsing started immediately: 5 mL for 15 s. Then immersed in solution for 15 min	Saline	Intermediate Restorative Material (IRM; Dentsply Sirona, Charlotte, NC, USA), MTA Angelus White (Angelus, Londrina, Brazil), Biodentine (Septodont, Saint-Maur-des-Fossés, Cedex, France), EndoCem Zr (Maruchi, Wonju, Republic of Korea), MTA HP (Angelus, Londrina, Brazil).	Evaluation under the microscope and depth map created using a 3D dental scanner (KaVo ARCTICA AutoScan).

**Table 4:** Washout of materials in the analyzed studies.

Author	Tested Material	Results	Conclusions
Jang et al., 2013 [21]	EndoCem (Maruchi, Republic of Korea), ProRoot MTA (Tulsa Dental Specialities, USA)	ProRoot showed higher washout scores than Endocem (*p* < 0.05).	Endocem is more resistant to washout than ProRoot
Smith et al., 2007 [22]	ProRoot white MTA (Dentsply Tulsa Dental, USA)	Irrigation with EDTA and BioPure MTAD resulted in only minor volume reductions of the set MTA.	ProRoot white MTA is not susceptible to washout
Porter et al., 2010 [23]	White MTA ProRoot (Tulsa Dental Specialities, USA), Capasio (Primus Consulting, USA), Ceramicrete-D (Tulsa Dental Specialities, USA), Generex-A (Dentsply, USA)	No material loss: Ceramicrete-D, Generex-A and Capasio Significant washout: White MTA ProRoot	White MTA ProRoot is more prone to washout than Ceramicrete-D, Generex-A and Capasio were that were resistant to rinsing
Grech et al., 2013 [15]	Tricalcium silicate cement (Mineral Research Processing, France) replaced with 20% zirconium oxide (Sigma–Aldrich, Germany), BiodentineTM (Septodont, France), BioaggregateT (Verio Dental, Canada),	Very low washout: tricalcium silicate cement, Bioaggregate and IRM high washout: Biodentine	Radiopacified tricalcium silicate, Bioaggregate and IRM exhibit low washout. Biodentine demonstrated a very high washout tendency.
Formosa et al., 2012 [24]	MTA-Plus (Avalon Biomed, India), Portland cement (PC, LaFarge Cement, UK), MTA-Angelus (Angelus, Brazil), IRM (Dentsply, Germany, amalgam (AB Ardent, Sweden)	Results expressed in mass loss: 0% IRM and amalgam, 0.9% MTA-AW (MTA with an antiwashout gel), 0.4–4% MTA-Plus, 2–7% PC, 5–10% MTA Angelus	The antiwashout gel used with MTA-Plus reduced the material washout and was similar to IRM and amalgam.
Falkowska et al., 2023 [25]	IRM (Dentsply, USA), MTA Angelus White (Angelus, Brazil), Biodentine (Septodont, France), EndoCem Zr (Maruchi, Republic of Korea), MTA HP (Angelus, Brazil).	No washout: IRM Very low washout: EndoCem MTA Zr and MTA HP Slightly greater washout: MTA Angelus White Significant washout: Biodentine	IRM, EndoCem MTA Zr and MTA HP showed good washout resistance. MTA Angelus White showed relatively good washout resistance. The Biodentine material was prone to washout, which is worrying and requires further research.

**Table 5:** Washout of materials.

Materials with Very Good Washout Resistance	EndoCem (Maruchi, Republic of Korea) [21,25]; Capasio (Primus Consulting, USA) [23]; Ceramicrete-D (Tulsa Dental Specialities, USA) [23]; Generex-A (Dentsply, USA) [23]; Bioaggregate (Verio Dental Co., Ltd., Canada) [15]; MTA-Plus (Avalon Biomed, India) [24]; MTA HP (Angelus, Brazil) [25]
Materials with Good Washout Resistance	ProRoot (Tulsa Dental Specialities, USA), [21,22,23]; MTA-Angelus [24]/MTA Angelus White (Angelus, Brazil) [25]
Materials Prone to Washout	Biodentine (Septodont, France) [15,25]

**Table 6:** Methods of washout evaluation.

Author	Evaluation	Unit of Measurement
Jang et al., 2013 [21]	SEM images	Images were evaluated using a developed scoring system. Percentage (%) of defect area.
Smith et al., 2007 [22]	3-D profilometry	The volume loss (mm^3^)
Porter et al., 2010 [23]	Photos (10×)	Marginal integrity of the materials was scored using photos to determine the percentage (%) of margin remaining.
Grech et al., 2013 [15]	Loss of mass of the sample calculated using Equation.	Percentage (%) washout by mass (g) of the test materials
Formosa et al., 2013 [24]	Loss of mass of the sample calculated using Equation.	Percentage (%) washout by mass (g) of the test materials
Falkowska et al., 2023 [25]	Evaluation under the microscope (60×) and depth map created using a 3D dental scanner (KaVo ARCTICA AutoScan).	Images were scored using a developed scoring system (1–3). Depth map: volume change (mm^3^ and %) and depth (mm)

## Data Availability

No new data were created or analyzed in this study.
